# Supplementary material for: DOPAnization of tyrosine in α-synuclein by tyrosine hydroxylase leads to the formation of oligomers
Source: Nat Commun. 2022 Nov 12;13:6880. doi: 10.1038/s41467-022-34555-4 (PMC9653393; doi:10.1038/s41467-022-34555-4)
Supplement: Supplementary file 1 — Supplementary information [file 41467_2022_34555_MOESM1_ESM.pdf]

Supplementary Materials for

DOPAnization of tyrosine in α-synuclein by tyrosine hydroxylase leads to the formation of oligomers

Mingyue Jin, Sakiko Matsumoto, Takashi Ayaki, Hodaka Yamakado, Tomoyuki Taguchi, Natsuko Togawa, Ayumu Konno, Hirokazu Hirai, Hiroshi Nakajima, Shoji Komai, Ryuichi Ishida, Syuhei Chiba, Ryosuke Takahashi, Toshifumi Takao, Shinji Hirotsune

Supplementary Figs. 1 to 9

Supplementary Table 1

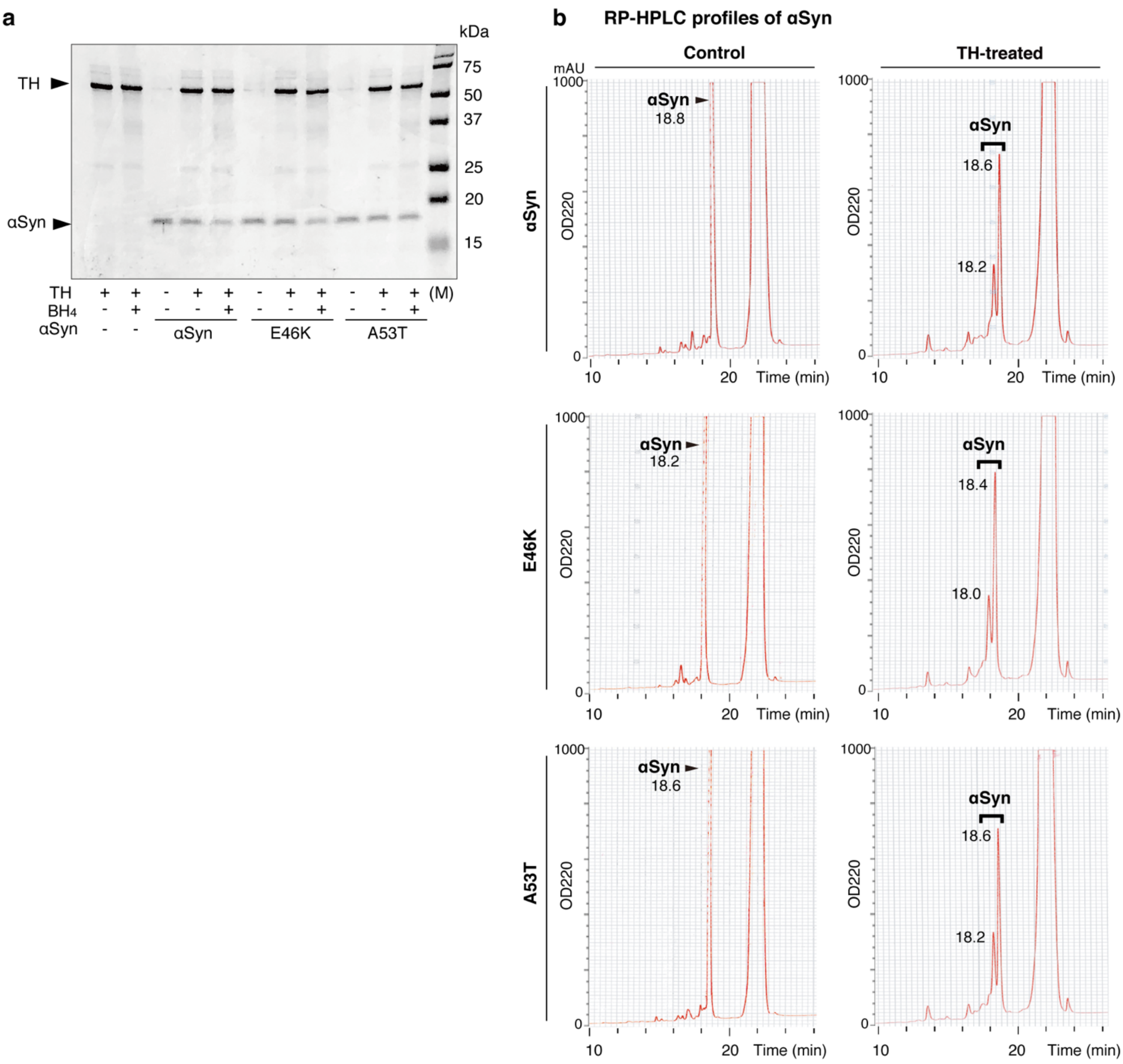

Supplementary Fig. 1: *In vitro* reaction of recombinant αSyn and TH.

a, SDS–PAGE after the *in vitro* reaction. Recombinant αSyn, E46K and A53T were incubated with or without TH and its cofactor, BH_4, and separated by SDS–PAGE. Proteins on the gel were stained with Coomassie brilliant blue. M: molecular marker. Data are representative of three independent experiments with similar results. **b,** RP-HPLC profiles of αSyn (top), E46K (middle) and A53T (bottom) with (TH-treated, right) or without (Control, left) TH treatment. GST-tagged αSyn, E46K and A53T were incubated with or without TH and purified by GST Sepharose beads. After removal of the GST tag, αSyn and mutants were separated by RP-HPLC and detected at 220 nm. Compared with the controls, the TH-treated samples had a new separation peak. The indicated αSyn fraction was used for subsequent trypsin digestion. The fractionation times (min) are indicated just above the peaks.

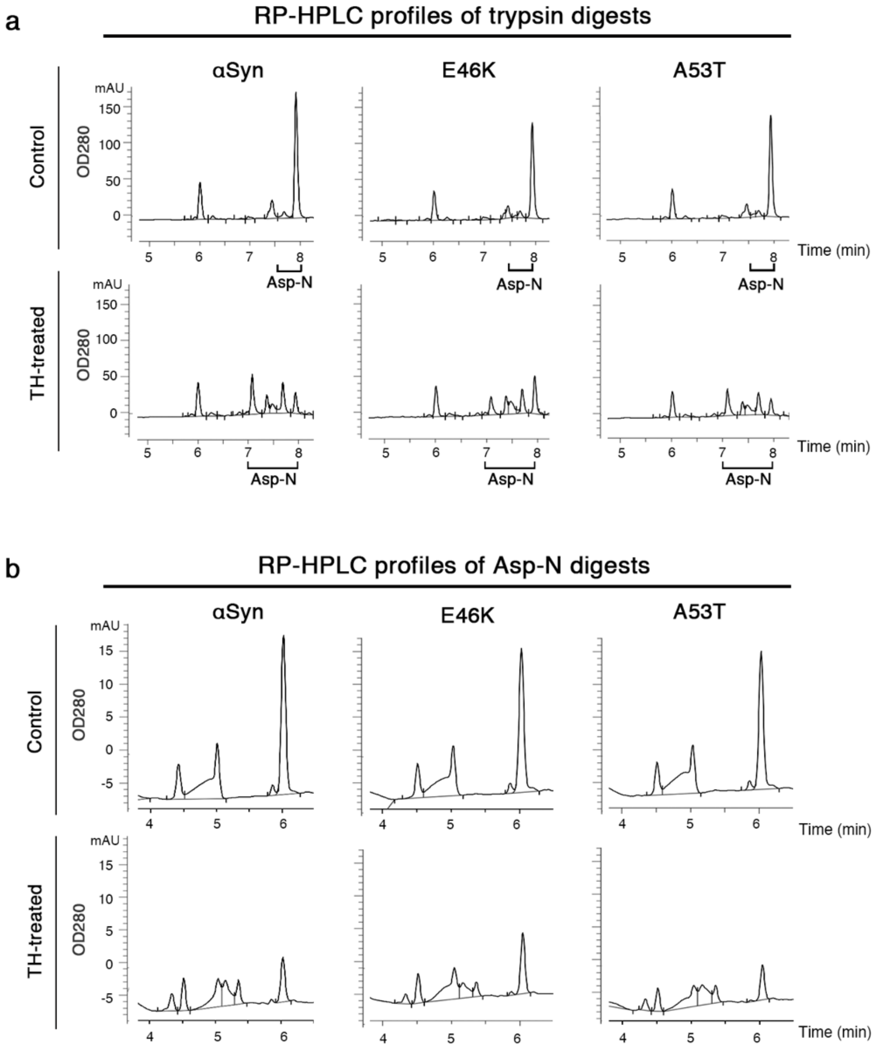

c

Correspondence of RP-HPLC fractions to αSyn fragments

	Trypsin HPLC RT	Asp-N HPLC RT	Mass [M+H]+	Sequence (Residues of αSyn)
Control	6.0-6.5		951.4	EGVLYVGSK (35-43)
	7.5-8.0	4.0-4.5	823.4	DPDNEAY (119-125)
			723.2	DYEPEA (135-140)
		4.5-5.0	1069.5	EMPSEEGYQ (126-134)
		6.0-6.5	1285.6	NEEGAPQEGILE (103-114)
TH-treated	6.0-6.5		951.4	EGVLYVGSK (35-43)
	7.0-8.0	4.0-4.5	823.4	DPDNEAY (119-125)
			723.2	DYEPEA (135-140)
			739.2	D-Dopa-EPEA (135-140)
		4.5-5.0	1069.5	EMPSEEGYQ (126-134)
		6.0-6.5	1285.6	NEEGAPQEGILE (103-114)

Supplementary Fig. 2: RP-HPLC separation and MS identification of digested peptides of αSyn.

a, RP-HPLC profiles of tryptic digests of αSyn. Tyrosine-containing peptides were detected at 280 nm, where tyrosine has a specific absorption. Compared with the controls (upper), the TH-treated samples resulted in the appearance of new peaks (lower). The indicated fractions were subjected to Asp-N digestion. **b,** RP-HPLC separation of C-terminal fractions (103-140 aa) derived from (a) subjected to Asp-N digestion. Similar to trypsin digestion, additional fraction peaks were also detected in the TH-treated samples (lower). Each 0.5-min fraction

was subjected to MS analysis. **c,** Correspondence of RP-HPLC elution to identified fragment sequences by MS/MS. The RP-HPLC fractions indicated by certain retention times (RT, min) were subjected to MS and MS/MS, and their amino acid sequences were determined. Tyrosine and Dopa residues were shown in red.

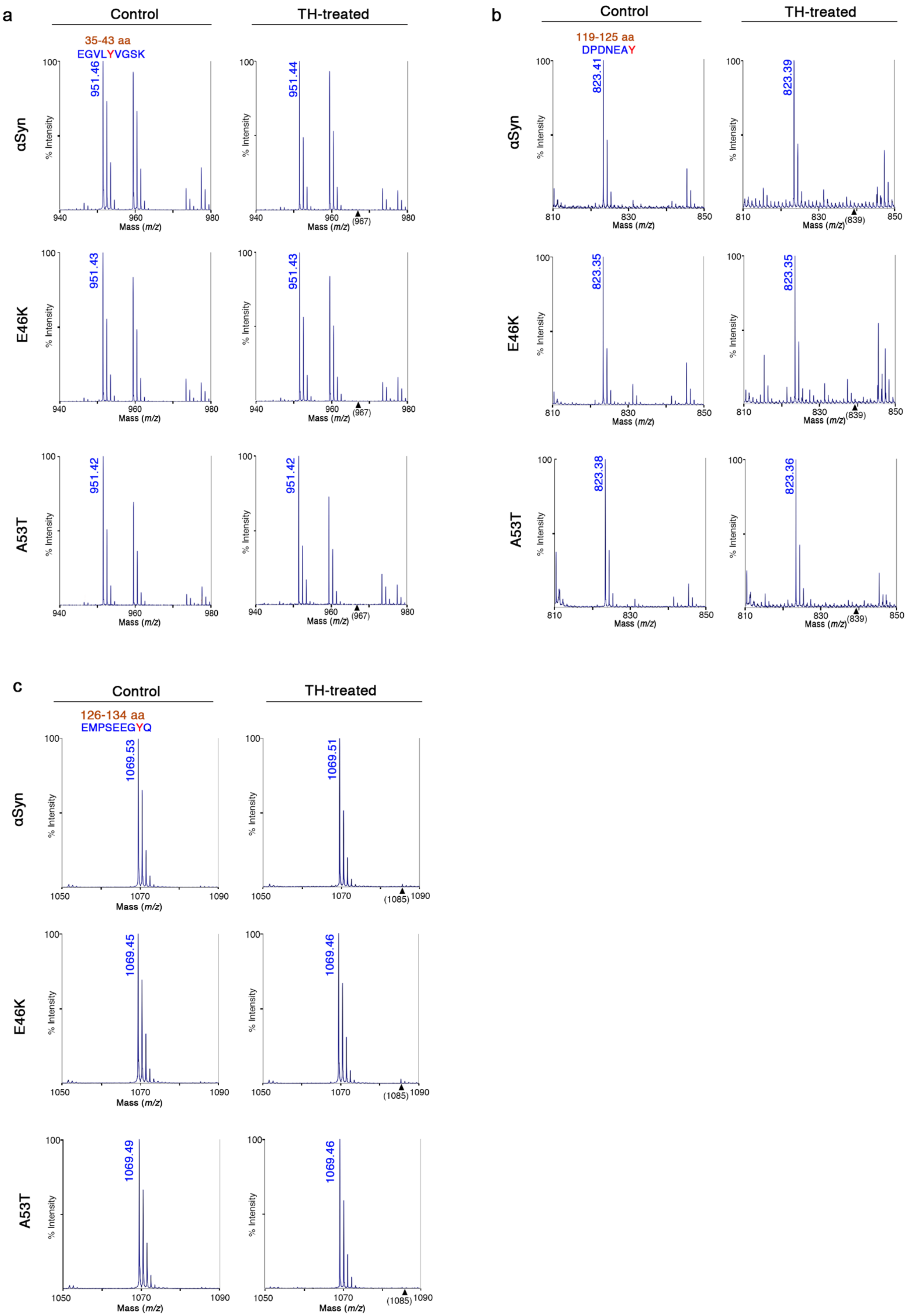
a
Control
TH-treated
35-43 aa
EGVLYVGSK
αSyn
E46K
A53T
% Intensity
Mass (m/z)
951.46
951.44
951.43
951.43
951.42
951.42
(967)
b
Control
TH-treated
119-125 aa
DPDNEAY
823.41
823.39
823.35
823.35
823.38
823.36
(839)
c
Control
TH-treated
126-134 aa
EMPSEEGYQ
1069.53
1069.51
1069.45
1069.46
1069.49
1069.46
(1085)

Supplementary Fig. 3: MS analysis of fragments containing Tyr39, 125 and 133 of αSyn.

a, Mass spectrum of the trypsin-digested fragment containing Tyr39 of αSyn, E46K or A53T. The peak at *m/z* 951.4 corresponds to 35-43 aa. There was no peak representing a +16 greater mass (*m/z* 967.4, black arrowheads) in the TH-treated samples. The peak at *m/z* 959.4 shows 35-43 aa fragment with artificial modifications. **b,** Mass spectrum of the fragment containing Tyr125 after Asp-N digestion. The peak at *m/z* 823.4 corresponds to 119-125 aa. There was no significant peak at *m/z* 839.4 after TH treatment (right, black arrowheads), indicating no oxidative modification at Tyr125. The peak at *m/z* 845.4 corresponds to sodium adduct form of the 119-125 aa. **c,** Mass spectrum of the fragment containing Tyr133 after Asp-N digestion. The peak at *m/z* 1069.5 corresponds to 126-134 aa. There was no significant peak at *m/z* 1085.5 after TH treatment (right, black arrowheads), indicating no oxidative modification at Tyr133 or Met127.

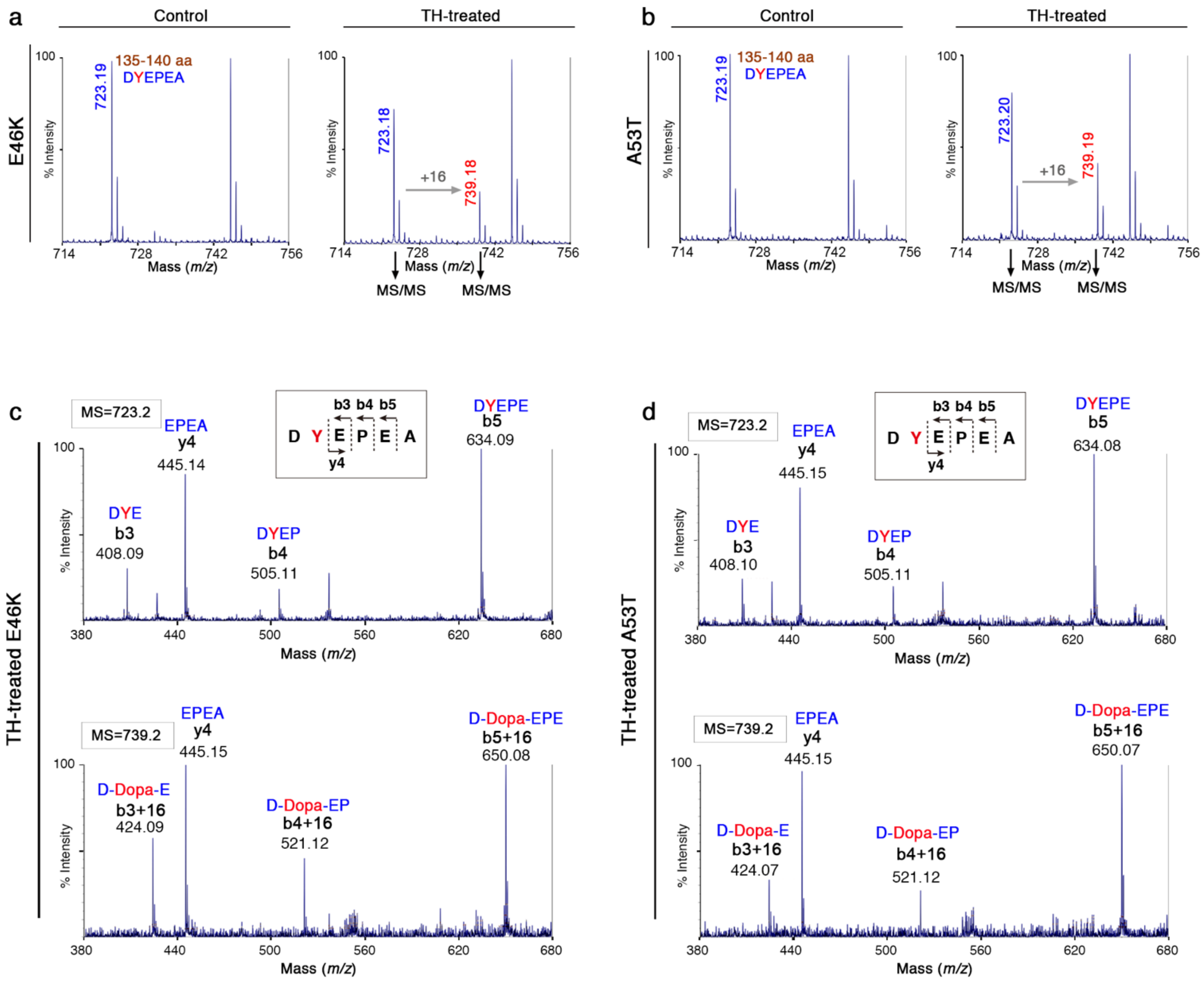

Supplementary Fig. 4: Identification of hydroxylation of αSyn mutants at Tyr136 by MS analysis.

a, b, MALDI-TOF mass spectrum of the fragment corresponding to 135-140 aa of E46K (**a**) and A53T (**b**). The peak at *m/z* 723.2 corresponds to 135-140 aa containing Tyr136 (left). An additional peak at *m/z* 739.2 appeared after TH treatment (right), showing a +16 greater mass than the 135-140 fragment. The peak at *m/z* 745.2 corresponds to sodium adduct form of the 135-140 aa. **c, d,** MALDI-TOF MS/MS spectra of the MS peaks at *m/z* 723.2 (upper) and *m/z* 739.2 (lower) from TH-treated E46K (**c**) and A53T (**d**). Fragmentation sites are indicated in

the boxed area. The peptide sequences of fragmentated ions are shown above each peak, identifying the site of dopanization as Tyr136.

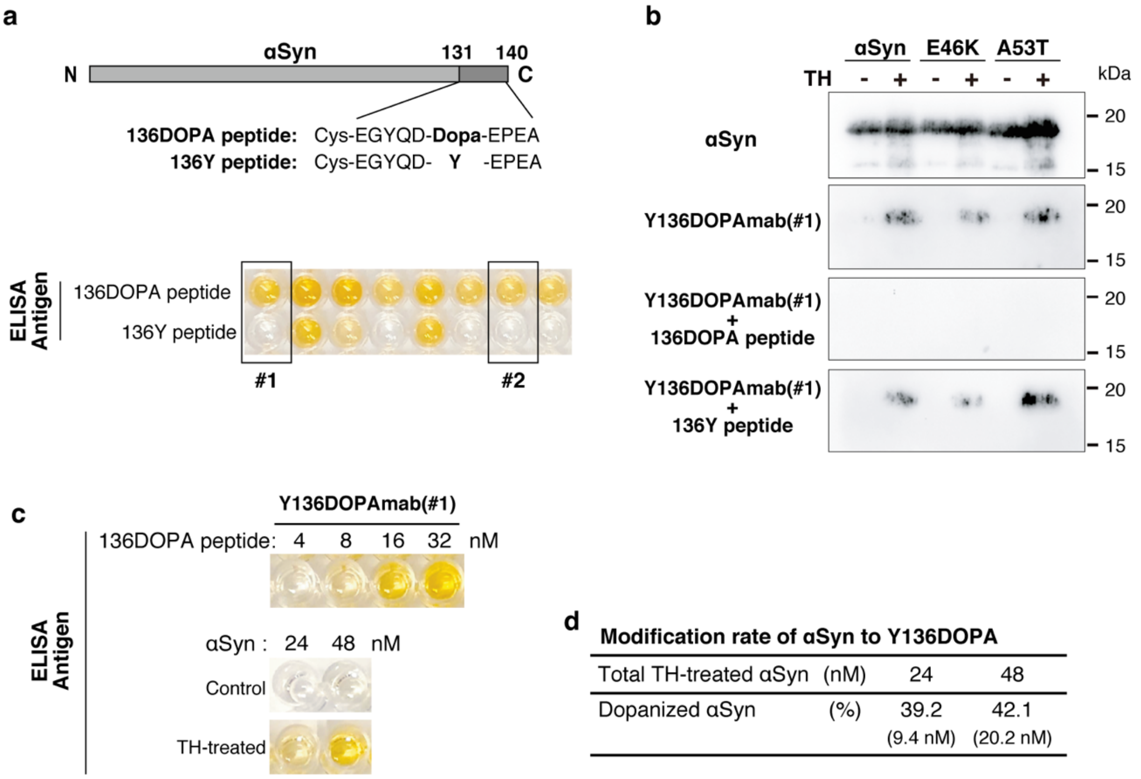

Supplementary Fig. 5: Generation of the Y136DOPA-specific antibody.

a, Generation of Y136DOPAmab. The design of synthetic peptides to produce a Y136DOPA-specific antibody is shown (upper). Cysteine (Cys) was conjugated at the N-terminal side of the peptide corresponding to 131-140 aa of αSyn. KLH-conjugated 136DOPA peptide was used for mouse immunization. The hybridoma screening was performed by ELISA (lower). Of more than 6000 clones, 135 clones represented 136DOPA peptide (+)/136Y peptide (-) in the ELISA test, and only two clones (#1 and 2) detected the Y136DOPA modification of αSyn in WB. **b,** Antibody absorption assay. Y136DOPAmab (#1) was preincubated with 136DOPA or 136Y peptide and subjected to WB analysis using control (TH-) or dopanized (TH+) αSyn and its mutants. 136DOPA, but not the 136Y peptide, specifically absorbed Y136DOPAmab. Data are representative of two independent experiments with similar results. **c, d,** Quantification of dopanized αSyn in the *in vitro* reaction. The proportion of Y136DOPA converted by TH *in vitro* was quantified by ELISA using Y136DOPAmab (**c**). Based on the absorbance (490 nm) from the 4-32 nM 136DOPA peptide, the amount of Y136DOPA in total αSyn (24 or 48 nM) used in the *in vitro* reaction was calculated (**d**). Approximately 40% of αSyn was dopanized by TH treatment.

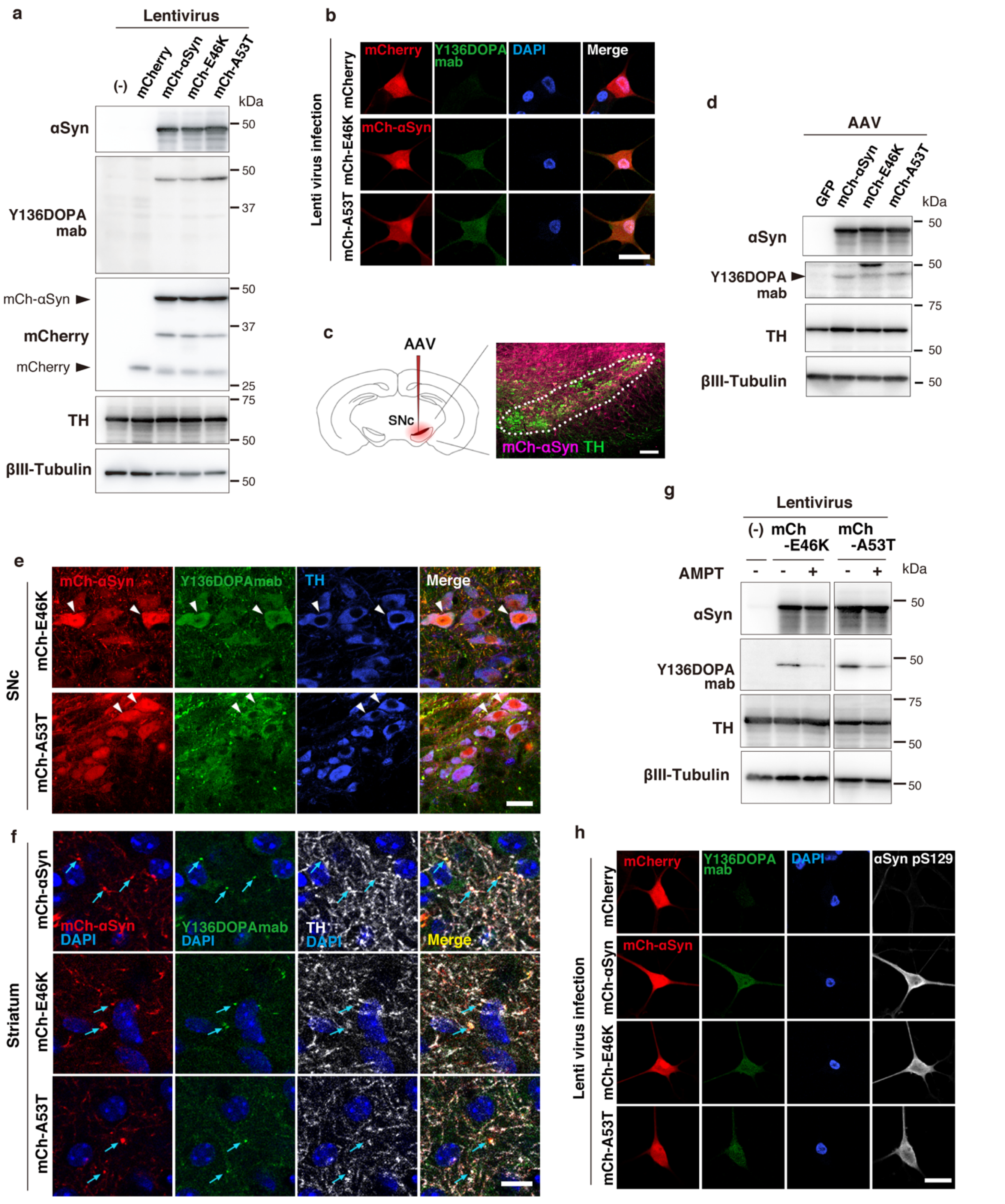

Supplementary Fig. 6: Y136DOPA modification of overexpressed mCh-αSyn *in vivo*.

a, Y136DOPA modification of mCh-αSyn in PC12 cells. mCherry, mCh-αSyn, mCh-E46K or mCh-A53T was overexpressed in PC12 cells by the lentivirus system. Cell lysates were extracted at Day 11 after infection and examined by WB. **b,** Y136DOPA signal detected by immunocytochemistry. PC12 cells overexpressing mCh-E46K (middle) or mCh-A53T (bottom), but not mCherry alone (upper), showed Y136DOPA signals (green) at Day 8 after lentivirus infection. Scale bar: 20 μm. **c,** Stereotaxic injection of AAV. Adult mice underwent unilateral injection of AAV expressing GFP or mCh-αSyn in the SNc (left). AAV-derived mCh-αSyn (magenta) was strongly expressed

in TH-positive dopaminergic neurons (green) in the SNc (right). Scale bar: 100 μm. **d,** WB analysis using AAV-injected SN tissues. Exogenously overexpressed mCh-αSyn was also dopanized by TH. **e, f,** Immunohistochemistry of AAV-injected brain sections. Y136DOPA modification (green) of mCh-αSyn (red) was detected in TH-positive dopaminergic neurons (blue in e) in the cell bodies of the SNc (**e**, white arrowheads), as well as TH-labeled nerve terminals of SNc neurons (white in f) in the striatum (**f**, blue arrows). Scale bars: 20 μm (e) and 10 μm (f). **g**, Y136DOPA signals were decreased by the TH inhibitor. PC12 cells overexpressing mCh-E46K and mCh-A53T at Day 10 after lentivirus infection were treated with 1 mM AMPT for 9 h and analyzed by WB. Y136DOPA signals were decreased by inhibiting endogenous TH activity. **h,** Double staining of phosphorylated Ser129 (pS129) and Y136DOPA of αSyn. Immunocytochemistry detected both Y136DOPA (green) and pS129 (white) signals of mCh-αSyn and its mutants in PC12 cells at Day 8 after lentivirus infection. Scale bar: 20 μm. Data are representative of two independent experiments with similar results (**a-h**).

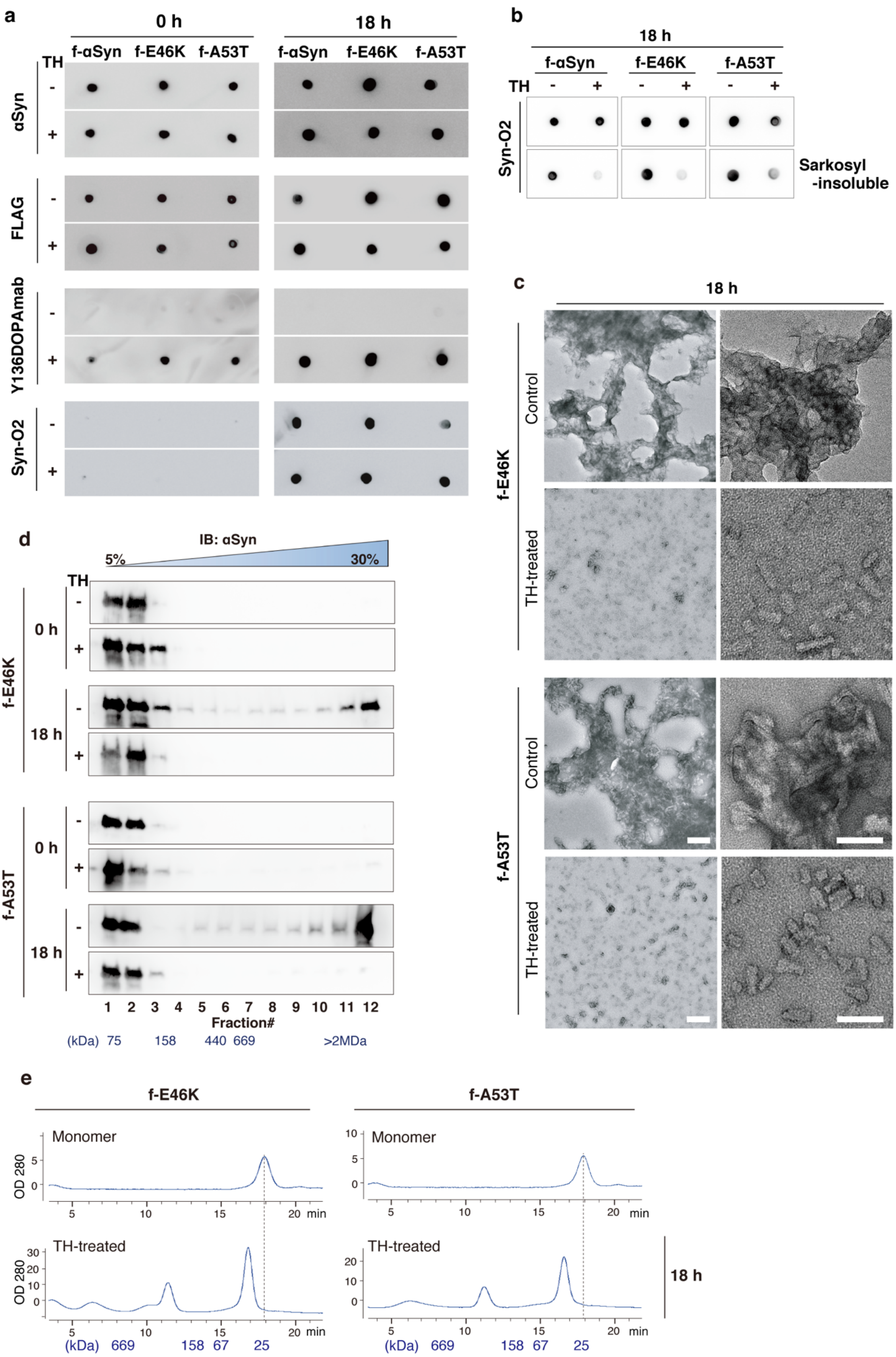

Supplementary Fig. 7: Fibrillization of f-αSyn.

a, Dot blot analysis of f-αSyn. Control (TH-) or TH-treated (TH+) f-αSyn, f-E46K and f-A53T were incubated for 18 h with continuous agitation. Before (0 h, left panel) or after (18 h, right panel) incubation, f-αSyn and two mutants

were spotted on nitrocellulose membranes and immunostained. An antibody that recognized both oligomeric and fibrillar forms of αSyn (Syn-O2) detected signals in both the control and dopanized f-αSyn only after incubation. Y136DOPAmab recognized TH-treated f-αSyn both before and after incubation. **b,** Solubility assay of f-αSyn. Control (TH-) and TH-treated (TH+) samples after 18 h of incubation (upper) were centrifuged and dissolved in 2% sarkosyl. The sarkosyl-insoluble aggregates (lower) were subjected to dot blot analysis using the Syn-O2 antibody. Control f-αSyn and two mutants included more sarkosyl-insoluble aggregates than the TH-treated f-αSyn. **c,** Negatively stained TEM images of mutated αSyn. Control and TH-treated f-E46K and f-A53T after 18 h of incubation were stained with 2% uranyl acetate. Control f-E46K and f-A53T formed large fibril clusters, whereas dopanized mutants formed a separated oligomeric conformation. High magnification images are shown on the right side of each panel. Scale bars: 200 nm (left) and 50 nm (right) **d,** WB analysis of the sucrose gradient fractionation of f-αSyn. Before (0 h) or after (18 h) 18-h incubation, f-E46K and f-A53T were separated by 5-30% sucrose gradient centrifugation. Twelve 1-mL fractions were examined by WB using an anti-αSyn antibody. The corresponding sucrose concentrations are indicated at the top of the panels. After 18 h of incubation, control f-αSyn formed large-mass aggregates, whereas dopanized f-αSyn retained a smaller mass. **e,** SEC of dopanized f-E46K and f-A53T oligomers. The chromatogram of SEC using monomeric (upper) or dopanized f-αSyn and two mutants after 18h of incubation (lower) showed that dopanized f-αSyn formed oligomers up to 15-mers. Data are representative of two independent experiments with similar results (**a-d**).

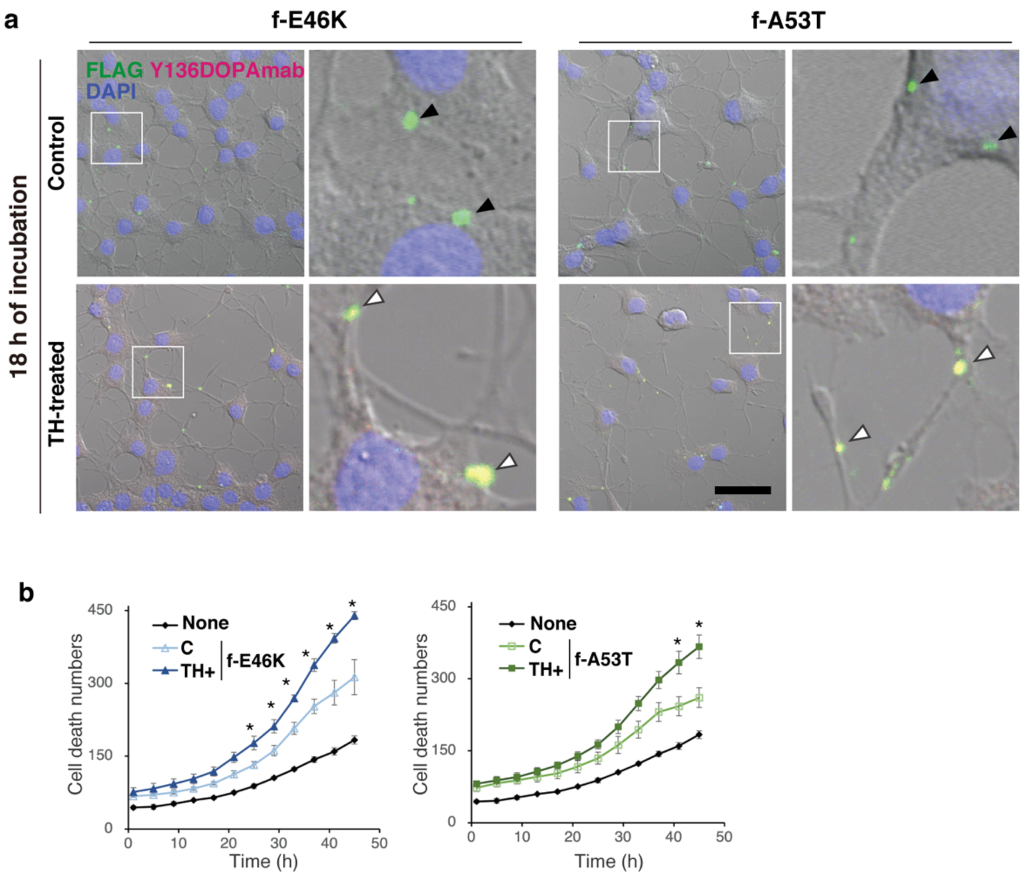

Supplementary Fig. 8: Cytotoxicity of dopanized f-αSyn mutants.

a, Detection of f-αSyn mutants uptake in PC12 cells. Control (upper) and dopanized (lower) f-E46K and f-A53T after 18 h of incubation were added to PC12 cells for 24 h and immunostained with anti-FLAG antibody (green)

and Y136DOPAmab (magenta). Rectangle-surrounding areas were enlarged on the right side. Black arrowheads indicate control samples taken up into cells, and white arrowheads point to dopanized samples (yellow). Scale bar: 20 μm. Data are representative of two independent experiments with similar results. **b,** Real-time quantification of cell death in PC12 cells treated with f-E46K (left) and f-A53T (right). The numbers of dead cells were counted using IncuCyte Cytotox Green Dye, showing a significant increase in cytotoxicity by treatment with the dopanized αSyn mutants (TH+) compared with that of the control αSyn mutants (C). Data are presented as mean ± SEM. Sample size: $n = 3$ well/treatment. *P*-values were calculated using two-tailed unpaired *t*-test between C and TH+ groups (f-E46K: 25 h $*p = 0.043$; 29 h $*p = 0.044$; 33 h $*p = 0.013$; 37 h $*p = 0.012$; 41 h $*p = 0.015$; 45 h $*p = 0.027$, f-A53T: 41 h $*p = 0.042$; 45 h $*p = 0.030$). None indicates the sample without any αSyn treatment. All samples, including wild-type f-αSyn (Fig. 4e), were simultaneously examined and are shown in separate graphs, where identical values of 'None' sample were used.

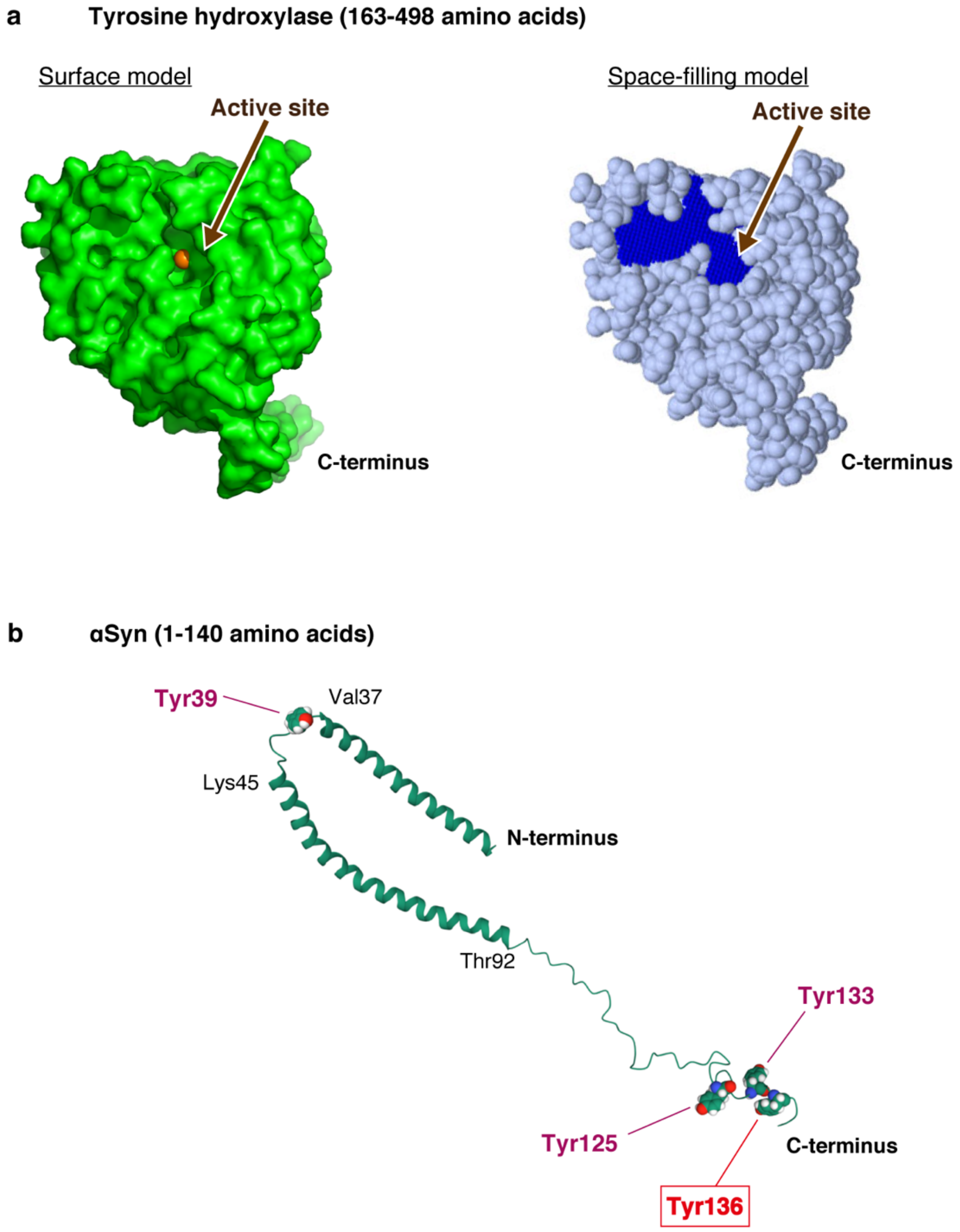

Supplementary Fig. 9: Structures of TH and αSyn.

a, Three-dimensional structure of rat TH (PDBID 1TOH). (Left) A surface model of TH (residues 163-498) is shown. A catalytic iron atom is indicated by an orange sphere. (Right) The cavity on the surface of TH was calculated with

GHECOM and displayed by Jmol. The TH structure is shown by the space-filling model. The blue-stained region indicates a groove containing the catalytic site, which a molecule with the maximum radius of 1.0 nm can access. **b,** Structure of full-length human αSyn (PDBID 1QX8) shown in a ribbon model. Tyrosine residues are highlighted by the space-filling model. Val3-Val37 and Lys45-Thr92 form curved α-helices, whereas Asp98-Ala140 forms a C-terminal acidic tail.

Supplementary Table 1: Case material studied.

Case	Diagnosis	Age (year)	Sex	Brain weight (g)	Duration of disease (year)	PMI (hours)
1	MSA-C	78	M	1100	2	2
2	MSA-C	72	F	1205	12	4
3	MSA-P	71	F	1105	2	3
4	PD	69	M	1400	9	12
5	PD	67	M	900	17	3
6	PD	78	F	1090	11	12
7	PD	77	M	1220	11	2
Sub-total		73.1 ± 4.5		1145.7 ± 153.3	9.1 ± 5.5	5.4 ± 4.5
8	FTLD	66	F	900		3
9	FTLD	64	M	1140		2.5
10	ALS	63	F	930		1.5
11	ALS	62	M	1290		1
12	CI	75	M	1110		6
13	VCI	86	F	940		4
14	GBS	62	M	1380		3
Sub-total		68.3 ± 9.0		1098.6 ± 187.4		3.0 ± 1.7

Abbreviations: PMI, postmortem interval; MSA-C, multiple system atrophy-cerebellar; MSA-P, multiple system atrophy-parkinsonian; PD, Parkinson disease; FTLD, frontotemporal lobar degeneration; ALS, amyotrophic lateral sclerosis; CI, cerebral infarction; VCI, vascular cognitive impairment; GBS, Guillain-Barré syndrome.

Sub total: Average ± SD.
