# Supplementary material for: DOPAnization of tyrosine in α-synuclein by tyrosine hydroxylase leads to the formation of oligomers
Source: Nat Commun. 2022 Nov 12;13:6880. doi: 10.1038/s41467-022-34555-4 (PMC9653393; doi:10.1038/s41467-022-34555-4)
Supplement: Supplementary file 3 — Reporting Summary [file 41467_2022_34555_MOESM3_ESM.pdf]

Corresponding author(s): Shinji Hirotsune
Last updated by author(s): Oct 18, 2022

Reporting Summary

Nature Portfolio wishes to improve the reproducibility of the work that we publish. This form provides structure for consistency and transparency in reporting. For further information on Nature Portfolio policies, see our Editorial Policies and the Editorial Policy Checklist.

Statistics

For all statistical analyses, confirm that the following items are present in the figure legend, table legend, main text, or Methods section.

n/a	Confirmed	
☐	☒	The exact sample size (*n*) for each experimental group/condition, given as a discrete number and unit of measurement
☐	☒	A statement on whether measurements were taken from distinct samples or whether the same sample was measured repeatedly
☐	☒	The statistical test(s) used AND whether they are one- or two-sided<br>*Only common tests should be described solely by name; describe more complex techniques in the Methods section.*
☒	☐	A description of all covariates tested
☒	☐	A description of any assumptions or corrections, such as tests of normality and adjustment for multiple comparisons
☐	☒	A full description of the statistical parameters including central tendency (e.g. means) or other basic estimates (e.g. regression coefficient) AND variation (e.g. standard deviation) or associated estimates of uncertainty (e.g. confidence intervals)
☐	☒	For null hypothesis testing, the test statistic (e.g. F, t, r) with confidence intervals, effect sizes, degrees of freedom and P value noted<br>*Give P values as exact values whenever suitable.*
☒	☐	For Bayesian analysis, information on the choice of priors and Markov chain Monte Carlo settings
☒	☐	For hierarchical and complex designs, identification of the appropriate level for tests and full reporting of outcomes
☒	☐	Estimates of effect sizes (e.g. Cohen's d, Pearson's r), indicating how they were calculated

Our web collection on statistics for biologists contains articles on many of the points above.

Software and code

Policy information about availability of computer code

Data collection	These softwares were used for data collection:<br>4000 series Explorer software (version 3.6; Applied Biosystems)<br>IncuCyte Base software (Sartorius)<br>Chemiluminescence imaging system Fusion solo S (VILBER)<br>Zeiss ZEN (Zeiss)
Data analysis	These softwares were used for data analysis:<br>Data Explorer software (version 4.8; Applied Biosystems)<br>Fiji (ImageJ ver. 1.53c; National Institutes of Health; https://fiji.sc/)<br>IncuCyte Base software (Sartorius)<br>GHECOM (http://strcomp.protein.osaka-u.ac.jp/ghecom/)<br>Jmol (http://www.jmol.org/)

For manuscripts utilizing custom algorithms or software that are central to the research but not yet described in published literature, software must be made available to editors and reviewers. We strongly encourage code deposition in a community repository (e.g. GitHub). See the Nature Portfolio guidelines for submitting code & software for further information.

Data

Policy information about availability of data

All manuscripts must include a data availability statement. This statement should provide the following information, where applicable:

- Accession codes, unique identifiers, or web links for publicly available datasets
- A description of any restrictions on data availability
- For clinical datasets or third party data, please ensure that the statement adheres to our policy

The data supporting the findings of this study are available in the manuscript and its supplementary information file. The TH and αSyn structure data used in this study are available in the Protein Data Bank under accession code PDB-1TOH [http://doi.org/10.2210/pdb1toh/pdb] and PDB-1XQ8 [http://doi.org/10.2210/pdb1xq8/pdb], respectively. The statistics data generated in this study are provided in the Source data file. Source data are provided with this paper.

Field-specific reporting

Please select the one below that is the best fit for your research. If you are not sure, read the appropriate sections before making your selection.

[x] Life sciences [ ] Behavioural & social sciences [ ] Ecological, evolutionary & environmental sciences

For a reference copy of the document with all sections, see nature.com/documents/nr-reporting-summary-flat.pdf

Life sciences study design

All studies must disclose on these points even when the disclosure is negative.

Sample size	No statistical test was performed to determine the sample size. The sample size follows common standards (n =3 biological replicates) and previously published work with comparable experimental setup. For postmortem brain tissue samples, the sample size was limited by the availability of patient numbers. Size is reported in legends for main and Supplementary figures.
Data exclusions	No data were excluded from the analysis.
Replication	All experiments were replicated at least twice independently. Biological replicates (at least 3) were described in the figure legend.
Randomization	Samples were allocated into experimental groups randomly.
Blinding	Blinding of the investigators was not relevant because the collected samples were equally treated and each experiment was performed under the same conditions. For MS analysis and cytotoxicity assay, data were automatically collected by softwares. The image analysis after immunohistochemistry was also automatically performed using software under the same parameter.

Reporting for specific materials, systems and methods

We require information from authors about some types of materials, experimental systems and methods used in many studies. Here, indicate whether each material, system or method listed is relevant to your study. If you are not sure if a list item applies to your research, read the appropriate section before selecting a response.

Materials & experimental systems

n/a	Involved in the study	
[ ]	[x]	Antibodies
[ ]	[x]	Eukaryotic cell lines
[x]	[ ]	Palaeontology and archaeology
[ ]	[x]	Animals and other organisms
[ ]	[x]	Human research participants
[x]	[ ]	Clinical data
[x]	[ ]	Dual use research of concern

Methods

n/a	Involved in the study	
[x]	[ ]	ChIP-seq
[x]	[ ]	Flow cytometry
[x]	[ ]	MRI-based neuroimaging

Antibodies

Antibodies used	TH (Rabbit polyclonal, Millipore, AB152); dilution rate: 1:1000; application: WB, IHC.<br>αSyn (Mouse monoclonal, BD Transduction Laboratories, 610787, 42/α-Synuclein); dilution rate: 1:1000; application: WB, dot blot assay.<br>RFP (Mouse monoclonal, MBL, M208-3, 1G9&3G5(mixed)); dilution rate: 1:2000; application: WB.<br>βIII-tubulin (Mouse monoclonal, R&D Systems, MAB1195, Tuj1); dilution rate: 1:2000; application: WB.<br>αSyn pS129 (Rabbit monoclonal, Abcam, ab168381, MJF-R13(8-8)); dilution rate: 1:1000; application: ICC<br>FLAG (Mouse monoclonal, Sigma, F1804, M2); dilution rate: 1:500; application: dot blot assay.

	FLAG (Rabbit monoclonal, Cell Signaling, 14793S, D6W5B); dilution rate: 1:200; application: ICC.<br>αSyn aggregated (Mouse monoclonal, BioLegend, 847602, Syn-O2); dilution rate: 1:500; application: dot blot assay.<br>Y136DOPAmab (Mouse monoclonal, hybridoma supernatant, generated in this study, 4B7); no dilution; application: WB, ELISA, ICC, IHC, dot blot assay.<br>Goat anti-Mouse IgG (H+L) Cross-Adsorbed Secondary Antibody, Alexa Fluor™ 488 (Goat polyclonal, Invitrogen, A11001); dilution rate: 1:1000 for ICC, IHC of mouse sections or 1:200 for IHC of human sections.<br>Goat anti-Rabbit IgG (H+L) Cross-Adsorbed Secondary Antibody, Alexa Fluor™ 488 (Goat polyclonal, Invitrogen, A11008); dilution rate: 1:1000 for ICC, IHC.<br>Goat anti-Mouse IgG (H+L) Cross-Adsorbed Secondary Antibody, Alexa Fluor™ 546 (Goat polyclonal, Invitrogen, A11003); dilution rate: 1:1000 for ICC.<br>Goat anti-Rabbit IgG (H+L) Cross-Adsorbed Secondary Antibody, Alexa Fluor™ 546 (Goat polyclonal, Invitrogen, A11010); dilution rate: 1:1000 for ICC, 1:200 for IHC of human sections.<br>Goat anti-Rabbit IgG (H+L) Cross-Adsorbed Secondary Antibody, Alexa Fluor™ 647 (Goat polyclonal, Invitrogen, A21244); dilution rate: 1:1000 for ICC, IHC.<br>Peroxidase AffiniPure Donkey Anti-Mouse IgG (H+L) (Donkey polyclonal, Jackson Immunoresearch, 715-135-150); dilution rate: 1:3000; application: WB, dot blot assay, ELISA.<br>Peroxidase AffiniPure Donkey Anti-Rabbit IgG (H+L) (Donkey polyclonal, Jackson Immunoresearch, 711-035-152); dilution rate: 1:3000; application: WB.
Validation	Validation details of the antibodies are available on the manufacturers' websites:<br>TH (https://www.merckmillipore.com/JP/ja/product/Anti-Tyrosine-Hydroxylase-Antibody,MM_NF-AB152)<br>αSyn (https://www.bdbiosciences.com/en-nz/products/reagents/microscopy-imaging-reagents/immunofluorescence-reagents/purified-mouse-anti-synuclein.610787)<br>RFP (https://ruo.mbl.co.jp/bio/dtl/A/?pcd=M208-3)<br>βIII-tubulin (https://www.rndsystems.com/products/neuron-specific-beta-iii-tubulin-antibody-tuj-1_mab1195)<br>αSyn pS129 (https://www.abcam.co.jp/alpha-synuclein-phospho-s129-antibody-mjf-r13-8-8-ab168381.html)<br>FLAG (https://www.sigmaaldrich.com/JP/ja/product/sigma/f1804?gclid=CjwKCAiAlrSPBhBaEiwAuLSDUCAaGi-lSWq5K5jjXtFiHKWj0y92G3xzr084rwO7ZhF7t9KJTkNScxoCumIQAvD_BwE)<br>FLAG (https://www.cellsignal.jp/products/primary-antibodies/dykddddk-tag-d6w5b-rabbit-mab-binds-to-same-epitope-as-sigma-s-anti-flag-m2-antibody/14793)<br>αSyn aggregated (relevant citation: Vaikath et al. (2015) Neurobiology of disease 79, 81-99)<br>Y136DOPAmab: the procedures of production and screening were described in the Methods of this paper. The specificity to Tyr136-dopanized αSyn was confirmed using WB and ELISA. Y136DOPA modification in recombinant and endogenous/exogenous human αSyn are recognized in human/mice brain and cultured cells.<br>Secondary antibodies conjugated with Alexa Flour 488, 546 or 647 (https://www.thermofisher.com/antibody/secondary/query/alexa)<br>Secondary antibodies conjugated with HRP (https://www.jacksonimmuno.com/technical/products/conjugate-selection/enzymes)

Eukaryotic cell lines

Policy information about cell lines

Cell line source(s)	Mouse myeloma cells (Sp2/O-Ag14, JCRB Cell Bank)<br>Lenti-X-293T cells (Takara)<br>PC12 cells (JCRB Cell Bank)
Authentication	Sp2/O-Ag14 cell line was subjected to isoezyme analysis and confirmed as mouse by NP, G6PD and MD.<br>PC12 cell line has not been authenticated.<br>Lenti-X 293T cell line was identified based on STR profiles by multiplex PCR.
Mycoplasma contamination	No contamination was confirmed by DAPI staining in the cell line of Lenti-X-293T and PC12 cells.<br>Myeloma cells were not tested for mycoplasma contamination.
Commonly misidentified lines<br>(See ICLAC register)	No commonly misidentified cell lines were used.

Animals and other organisms

Policy information about studies involving animals; ARRIVE guidelines recommended for reporting animal research

Laboratory animals	C57BL/6 mice were used (male and female, post natal day 0-2 (n = 20), 9-15 weeks (n = 16) and 18 months old (n = 3)).<br>A53T BAC transgenic mice were maintained in C57BL/6 background, and male and female mice of 18 months old (n = 3) were used.<br>Female BALB/c mice of 7 weeks old (n = 20) were used for the generation of monoclonal antibody.
Wild animals	No wild animals were used in this study.
Field-collected samples	No field-collected samples were used in this study.
Ethics oversight	All the animal experiments were performed in accordance with the institutional guideline for animal experiments approved by the Institutional Animal Care and Use Committee, Osaka Metropolitan University (approval number #21094) and Kyoto University (approval number #Med Kyo 21001).

Note that full information on the approval of the study protocol must also be provided in the manuscript.

Human research participants

Policy information about studies involving human research participants

Population characteristics	Information for the participants was provided in Supplementary Table 1.<br>MSA and PD:<br>1. Age: 78 year-old, Gender: M, Diagnosis: MSA, Disease duration: 2 years, PMI: 2 hours<br>2. Age: 72 year-old, Gender: F, Diagnosis: MSA, Disease duration: 12 years, PMI: 4 hours<br>3. Age: 71 year-old, Gender: F, Diagnosis: MSA, Disease duration: 2 years, PMI: 3 hours<br>4. Age: 69 year-old, Gender: M, Diagnosis: PD, Disease duration: 9 years, PMI: 12 hours<br>5. Age: 67 year-old, Gender: M, Diagnosis: PD, Disease duration: 17 years, PMI: 3 hours<br>6. Age: 78 year-old, Gender: F, Diagnosis: PD, Disease duration: 11 years, PMI: 12 hours<br>7. Age: 77 year-old, Gender: M, Diagnosis: PD, Disease duration: 11 years, PMI: 2 hours<br>Control:<br>1. Age: 66 year-old, Gender: F, Diagnosis: frontotemporal lobar degeneration, PMI: 3 hours<br>2. Age: 64 year-old, Gender: M, Diagnosis: frontotemporal lobar degeneration, PMI: 2.5 hours<br>3. Age: 63 year-old, Gender: F, Diagnosis: ALS, PMI: 1.5 hours<br>4. Age: 62 year-old, Gender: M, Diagnosis: ALS, PMI: 1 hour<br>5. Age: 75 year-old, Gender: M, Diagnosis: cerebral infraction, PMI: 6 hours<br>6. Age: 86 year-old, Gender: F, Diagnosis: vascular cognitive impairment, PMI: 4 hours<br>7. Age: 62 year-old, Gender: M, Diagnosis: Guillain-Barre syndrome, PMI: 3 hours
Recruitment	Postmortem brain tissues from disease and control cases were supplied by Kyoto University hospital. Clinically and pathologically examined brain samples without age difference and other complications have been chosen in both control and disease cases. Although these samples are considered to represent the expected population in each group, the limited, single centered samples may cause a potential selection bias.
Ethics oversight	The study using postmortem brain samples from human subjects was approved by the Ethics Committee of Kyoto University (approval number #R1038).

Note that full information on the approval of the study protocol must also be provided in the manuscript.
